# The Heel-Strike Fallacy and Cadence Beliefs in Distance Running: A Narrative Review

**DOI:** 10.1186/s40798-026-01120-y

**Published:** 2026-10-07

**Authors:** Aurélien Patoz, Cyrille Gindre, Thibault Lussiana, Bastiaan Breine

**Affiliations:** 1 Research and Development Department, Volodalen SwissSportLab, 1023 Crissier, Switzerland; 2 Institute of Sport Sciences, University of Lausanne, 1015 Lausanne, Switzerland; 3 Research and Development Department, Volodalen, 39270 Chavéria, France; 4 Université Marie et Louis Pasteur, SINERGIES, 25000 Besançon, France; 5 Plateforme Exercice Performance Santé Innovation, Université Marie et Louis Pasteur, 25000 Besançon, France; 6 Department of Movement and Sports Sciences, Ghent University, 9000 Ghent, Belgium

**Keywords:** Running myth, Gait retraining, Foot-strike pattern, Cadence, Duty factor, Musculoskeletal loading, Overuse injury

## Abstract

Running-technique recommendations often convert group-level observations and acute biomechanical responses into universal prescriptions. This narrative review examines three beliefs: rearfoot striking is inherently harmful, 180 steps/min is universally optimal, and increasing cadence while maintaining a given running speed consistently reduces loading and overuse injury risk. Current evidence does not support these generalizations. First, rearfoot striking is not consistently associated with greater impact-related metrics or overuse injury risk, and external impact measures do not directly represent tissue-specific loading. Second, cadence varies substantially with running speed, morphology, footwear, and surface, while energetically optimal cadence varies between individuals rather than being fixed at 180 steps/min. Third, although increasing cadence can acutely modify selected mechanical variables, these responses are heterogeneous, have not been shown to consistently reduce injury risk, and may partly reflect concurrent changes in stance–flight organization. Duty factor (DF), the proportion of the stride spent in contact with the ground, provides a complementary temporal descriptor that can help interpret these cadence-related responses. Under level, submaximal running conditions, DF is associated with selected external and modeled internal loads, but current evidence is predominantly mechanistic. Clinical application should therefore begin by determining whether change is warranted, identifying the tissue-specific or functional objective, and selecting whether and how to modify foot-strike pattern or cadence according to condition-specific evidence and the runner’s response. DF may additionally be monitored to characterize accompanying changes in stance–flight organization. Acute biomechanical changes should not be assumed to improve symptoms or performance, or to reduce injury risk. Future studies should test sustained clinical effects, individual response patterns, and transfer to real-world conditions.

## Highlights

- Rearfoot striking is not inherently harmful. Rearfoot striking is not consistently associated with greater impact-related loading or overuse injury risk, and external impact measures should not be interpreted as direct indicators of tissue-specific loading.
- Cadence is context-dependent rather than universally optimal. Cross-sectional evidence shows that cadence varies substantially with running speed, morphology, footwear, and surface, and no fixed value such as 180 steps/min represents a universal optimum.
- Habitual running form should generally be preserved unless change is warranted. Foot-strike pattern and cadence should not be modified solely to conform to generalized biomechanical targets; modification should be considered when a tissue-specific, symptom-related, functional, or performance rationale exists and should be evaluated according to the individual runner’s response.

## Background

Running technique is frequently targeted in attempts to improve performance, modify mechanical loading, or manage running-related overuse injuries. Foot-strike modification and cadence manipulation have received substantial attention in biomechanical research and running gait retraining [1–5]. Their practical appeal lies in offering observable and quantifiable variables that can be readily manipulated. However, a measurable biomechanical response does not establish that the same intervention improves performance, symptoms, or injury-related outcomes across runners and task conditions [2, 6–10].

A central challenge in translating running biomechanics into practice is the distinction between different levels of evidence. For instance, ground-reaction forces and loading rates characterize external ground interaction, whereas tibial accelerations describe the segmental response to impact and are frequently used as indirect loading indicators; however, these measures do not directly quantify tissue-specific musculoskeletal loading and may correlate only weakly with modeled internal forces [6, 7, 11–14]. Similarly, an acute change in a biomechanical variable does not by itself demonstrate improved running economy, reduced pain, or a lower risk of future injury [1, 2, 8, 15]. External mechanics, internal musculoskeletal loading, performance, symptoms, and injury occurrence represent distinct levels of analysis, and findings at one level cannot be directly extrapolated to another [2, 6, 7, 9, 11].

This translational problem is apparent in the historical development of influential recommendations concerning running technique. Foundational biomechanical studies characterized impact transients associated with rearfoot striking [16–18]. Early clinical accounts proposed that the accumulation of impact loading could contribute to running-related overuse injury [19], while later impact-related frameworks further developed the proposed relationship between repetitive mechanical loading and overuse injury development [20]. Retrospective findings subsequently associated rearfoot striking with higher injury rates in a specific population of competitive runners [21]. In parallel, observations in competitive runners contributed to the recognition of cadence values near 180 steps/min [22]. Experimental studies subsequently showed that acute cadence manipulation could modify selected kinematic, kinetic, and muscle-activation variables [23–27], as later synthesized in systematic reviews [2, 28]. These studies provided important insights into running mechanics. Their subsequent interpretation and translation into gait-retraining recommendations are reflected in later reviews and applied publications concerning foot-strike modification and cadence manipulation [1–5, 29].

Contemporary evidence indicates that the relationships underlying these recommendations are more conditional and context dependent than generalized interpretations imply [1, 2, 6, 30–32]. Recent systematic reviews have identified persistent uncertainty regarding whether biomechanical changes induced by foot-strike or cadence modification translate into improved performance, sustained clinical benefits, or reduced injury risk [1, 2, 4, 10]. This uncertainty concerns the generalizability of these interventions and should not be interpreted as evidence that foot-strike or cadence modifications are never appropriate for individual runners [1, 2, 4, 10].

Foot-strike pattern and cadence vary with running speed, morphology, footwear, and surface [33–40], while evidence in adolescent runners further indicates that cadence differs according to physical maturation, even when running speed is accounted for [41]. Their associations with running economy, mechanical loading, and injury remain heterogeneous across individuals and study conditions [1, 2, 6, 30–32, 42]. Running technique is therefore increasingly understood as an emergent product of interacting individual, task, and environmental constraints [43, 44] rather than as a collection of independent variables with universally optimal values [45–48]. Importantly, what constitutes an “optimal” movement solution also depends on the outcome being optimized: predictive simulations have shown that different foot-strike patterns emerge when different mechanical or energetic cost functions are minimized [49]. Within this perspective, different coordination patterns may represent viable solutions to the same task, and deviation from a group mean does not necessarily indicate technical error or dysfunction [31, 45, 46, 48, 50–52]. Group-level studies remain essential for identifying average associations and intervention effects. The concern arises when a group mean is interpreted as an optimal target for every runner, because preferred coordination patterns and responses to biomechanical manipulation may differ among individuals and task conditions [34, 50–52]. In this review, individual differences refer primarily to between-runner differences in coordination and to context-dependent responses within a runner. This emphasis is conceptually related to, but distinct from, the optimal movement variability framework, which concerns the amount and temporal structure of fluctuations across repeated movements and their potential relationship with adaptability and pathology [53].

Against this background, the present review critically examines three beliefs in running biomechanics. These beliefs were selected a priori because they recur in systematic reviews and the applied gait-retraining literature [1–5, 29] and address variables that can be readily manipulated in practice.

1. **The heel-strike fallacy**—the belief that rearfoot striking is inherently harmful because it produces greater impact loading and overuse injury risk.
2. **The universal cadence rule**—the belief that near 180 steps/min represents an optimal cadence across runners.
3. **The cadence prescription**—the belief that systematically increasing cadence while maintaining a given running speed consistently reduces mechanical loading and overuse injury risk.

Together, these beliefs represent three distinct manifestations of the same translational problem: an observable movement pattern may be assigned an inherently harmful status, a descriptive group value may become a normative target, and an acute biomechanical response may be converted into a generalized intervention. Examining these beliefs together provides a coherent basis for distinguishing descriptive associations from causal inferences, and acute biomechanical responses from evidence of performance or clinical benefit [2, 54–56].

Evaluation of the third belief requires considering cadence within the broader temporal organization of running. Cadence describes the frequency of successive steps but does not indicate how stride time is distributed between stance and flight. Duty factor (DF)—the proportion of the stride spent in contact with the ground—is therefore considered here as a complementary temporal descriptor. Its purpose in this review is primarily to help determine whether mechanical effects commonly attributed to cadence may instead, or additionally, reflect concurrent changes in stance–flight organization. Cadence and DF represent distinct but interacting descriptors of running organization [48, 57]. Experimental work has shown that DF can be modulated through stance- or flight-time instructions without clear concomitant changes in cadence during treadmill running at a given speed [58], while subsequent field-based work showed that cadence and DF can also be jointly manipulated during overground running, although with greater co-variation in speed and the non-targeted variable [59]. Evidence from our group further demonstrates that cadence and DF can be independently manipulated across a functional range during endurance running [60]. Together, these findings support treating cadence and DF as related but distinguishable descriptors of running organization. Several experimental and observational studies conducted primarily during level, submaximal running have additionally associated DF with external force profiles and modeled internal musculoskeletal loading [61–64]. These findings provide a mechanistic lens through which the cadence prescription can be interpreted, without implying that DF itself has a universally beneficial value or direction of modification.

By tracing the origins of these beliefs and synthesizing the evidence supporting or challenging them, this narrative review aims to clarify the limits of universal recommendations. Foot-strike pattern and cadence provide a focused set of examples through which the translation of biomechanical observations into technical recommendations can be examined. They are considered here as observable descriptors embedded within a broader coordination system, rather than as isolated determinants or an exhaustive representation of running technique. Particular attention is given to distinctions among external mechanical measures, internal musculoskeletal loading, performance outcomes, and running-related overuse injury. The review also considers how individual differences and constraint-dependent coordination may inform the interpretation and application of biomechanical evidence, before outlining implications for clinical practice, coaching, and future research.

## Methods

This study employed a narrative review methodology [65] to examine the scientific and applied literature on running biomechanics and its translation into coaching and clinical practice. We reviewed empirical and conceptual publications addressing three key domains—foot-strike pattern and impact loading, running cadence, and cadence modification during gait retraining—to evaluate the origins, supporting evidence, persistence, and practical implications of three pervasive biomechanical beliefs.

The review was informed initially by recent systematic reviews of running biomechanics, running economy, and running-related overuse injury risk [32, 66]. These sources were used to identify key areas of uncertainty, influential studies, and relevant terminology for subsequent searches across scientific databases.

A structured literature search was conducted in PubMed, Scopus, and Web of Science for English-language publications available up to June 2026. Search terms were combined using the Boolean operators AND and OR and included “running” together with terms related to the three thematic areas: “foot strike”, “rearfoot strike”, “forefoot strike”, “impact”, “loading rate”, “cadence”, “step rate”, “stride frequency”, “180 steps per minute”, “gait retraining”, “duty factor”, “contact time”, “internal loading”, “running economy”, and “injury”. Search syntax was adapted to the requirements of each database. Reference lists of relevant systematic reviews and included articles were also screened to identify additional studies.

Peer-reviewed systematic reviews, intervention studies, prospective and retrospective studies, cross-sectional biomechanical studies, modeling studies, and relevant conceptual articles were considered. Articles were included when they directly addressed foot-strike patterns, cadence, cadence manipulation, DF, mechanical loading, running economy, or running-related overuse injury. Studies unrelated to running or not directly relevant to the three predefined themes were excluded.

Titles and abstracts were screened for relevance to the predefined review questions, followed by full-text assessment of potentially eligible publications. The identified literature was then organized into three thematic sections. All authors contributed to study selection and evidence synthesis. To reduce selective citation, study relevance was assessed according to the predefined scope and eligibility criteria rather than whether the findings supported or challenged the beliefs examined. Disagreements regarding study inclusion, interpretation, or the strength of a conclusion were resolved through discussion and consensus.

Evidence was interpreted according to study design and outcome type, distinguishing external biomechanical measures, internal musculoskeletal loading, and clinical outcomes. Greater weight was given to systematic reviews, prospective studies, intervention studies, and direct clinical outcomes, whereas acute biomechanical and modeling studies were primarily used to support mechanistic interpretations. No unpublished data were included.

## Discussion—The Three Pervasive Biomechanical Beliefs

### Belief 1: The Heel-Strike Fallacy

#### Definition and Historical Context

The first enduring belief asserts that heel striking is inherently detrimental because it generates higher impact forces that directly increase overuse injury risk. This assumption—often simplified to “heel striking causes injuries”—arose from early laboratory studies showing larger vertical impact peaks in rearfoot than in forefoot strikers [16, 17, 20, 21]. Force–time curves with sharp impact transients appeared hazardous, reinforcing the belief that non-rearfoot running was safer. In the 2000s, barefoot and minimalist movements—such as Pose and Chi Running—amplified this narrative [67–69]. Further popularized by McDougall [70] and influential research on habitually barefoot populations [17, 71], these movements reinforced the view—across both coaching and consumer contexts—that avoiding heel strike was essential for preventing injury and improving running economy.

Yet this narrative is difficult to reconcile with observed running behavior. Most endurance runners naturally adopt a rearfoot strike, with studies reporting this pattern in at least 55% of elite and 90% of recreational marathon runners [72–74]. Consistent with these observations, Van den Berghe, Warlop, Derie, Leman, De Clercq and Breine [75] identified a rearfoot strike in 92% of analyzed foot contacts in a cohort of 104 distance runners using high-speed, high-resolution plantar-pressure measurements. Its prevalence across runners with different morphologies, footwear, and training histories indicates that rearfoot striking represents a common movement solution rather than an inherently maladaptive one. Its spontaneous and stable adoption over prolonged exposure is consistent with the idea that running gait self-organizes through repeated practice toward solutions that balance energetic cost, mechanical loading, and comfort within individual constraints [30, 76].

#### Empirical Evidence: Rearfoot Striking Is Not Consistently Associated with Greater External Impact-Related Metrics

In this section, impact-related measures refer collectively to distinct external or segmental variables characterizing early stance, including the vertical impact peak, vertical loading rate, and tibial acceleration. Because these measures quantify different mechanical phenomena, the specific variable examined is identified wherever possible.

Contrary to this long-standing assumption, rearfoot striking is not consistently associated with higher external impact-related metrics, and current evidence does not establish that it increases overuse injury risk [1, 6, 77]. Cross-sectional analyses reveal substantial differences across individuals and foot-strike angles. The observed relationships also vary with running speed, footwear, the methods used to classify foot-strike pattern or angle, and the specific impact-related metric examined [35–38, 72–74, 76, 78–82]. For vertical loading rate, a curvilinear association with foot-strike angle has been reported [38, 78–81]. Across independent datasets, mild rearfoot strikers (approximately 5–10°) displayed higher vertical loading rates, whereas lower values have been observed in some pronounced rearfoot and forefoot strikers [38, 78–81]. Although these independent analyses broadly converge on a curvilinear association, differences in foot-strike descriptors, loading-rate definitions, running conditions, and fitted models limit direct quantitative comparison of the reported curves. This pattern is also illustrated by a case study of an exceptionally high-mileage runner who displayed a pronounced rearfoot strike angle (29°) together with a peak vertical loading rate 57% lower than that of matched rearfoot-striking controls [83]. In an additional analysis of 52 distance runners, the same study reported a quadratic relationship between foot-strike angle and peak vertical loading rate across multiple running speeds, with lower loading rates toward both ends of the foot-strike-angle continuum [83]. The curvilinear pattern should not be generalized to other impact-related metrics; for example, peak three-dimensional segmental accelerations across foot-strike patterns have shown different relationships [82].

These findings indicate that relatively low vertical loading rates can occur across different foot-strike patterns and angles rather than being exclusive to a particular strike category. However, foot-strike classification is continuous and method-dependent, and the supporting evidence remains predominantly cross-sectional [38, 78–81]. These observations therefore do not establish discrete protective strategies, causal effects, or differences in overuse injury risk.

A recent review concluded that vertical loading rate alone is insufficient to establish causal mechanisms for running-related injury [6]. Furthermore, the relationship between foot-strike pattern and overuse injury risk remains undetermined, as the available evidence is largely derived from retrospective analyses rather than prospective studies [1, 77]. Cross-sectional associations between foot-strike pattern and external loading should therefore not be interpreted as evidence of differences in internal tissue loading [7, 11] or future overuse injury risk [9].

Overall, the available evidence does not support inferring overuse injury risk from the point of ground contact alone. Rather than being determined by foot-strike pattern, overuse injury risk emerges from interactions among external mechanics, neuromuscular control, tissue adaptation, training exposure, and task conditions [30, 84–88].

#### Conceptual Clarification: External Impact Differs from Internal Load

The persistence of the heel-strike belief also stems from conflating external ground-reaction forces with internal tissue loading, which includes the forces and stresses experienced by musculoskeletal structures and depends in part on muscle forces. These internal contributions have historically received less attention than kinematic and ground-reaction-force analyses [6]. Modeling and experimental work have shown that impact transmission is modulated by neuromuscular control, joint kinematics, and soft-tissue damping [89, 90]. Gruber, Boyer, Derrick and Hamill [91] demonstrated that although forefoot and rearfoot runners display distinct impact-shock profiles, the attenuation of these vibrations through the kinetic chain is comparable, showing that distinct external impact profiles do not necessarily result in proportionally different vibration transmission through the body.

Prospective studies and systematic reviews do not generally support isolated external biomechanical measures as robust predictors of running-related overuse injury occurrence [9, 92]. Experimental and modeling studies further indicate that commonly used external and segmental impact-related measures, including ground-reaction-force metrics and tibial acceleration, may poorly represent internal tissue loading [7, 11, 13, 14]. This dissociation arises because internal loading also depends substantially on muscle forces, joint geometry, tissue properties, and the distribution of forces across the kinetic chain.

Recent musculoskeletal modeling in habitual rearfoot strikers showed that imposing a non-habitual forefoot strike increased distal tibial loading compared with both habitual and imposed rearfoot-strike conditions [93]. This finding illustrates that adopting a non-habitual forefoot strike does not necessarily reduce internal bone loading, even when the external impact profile is altered. An intervention study further shows that substantial reductions in tibial acceleration may occur without corresponding reductions in peak vertical ground-reaction force or peak joint moments [94]. This dissociation illustrates that impact-related metrics are not interchangeable with measures of internal tissue loading and that reducing a single feedback target should not be assumed to produce parallel changes in other mechanical variables or clinical outcomes.

Separate musculoskeletal and finite-element modeling studies further indicate that internal bone loading is sensitive to both task conditions and the temporal organization of running. Tibial strain increased with running speed but not with running grade [95]. In a subsequent study conducted at a fixed speed of 2.2 m/s, guided reductions in flight time were associated with lower peak tibial and fibular strains and a smaller volume of highly strained bone [96]. Together, these findings reinforce that internal bone loading depends on the interaction between running speed, stance–flight organization, and the broader coordination strategy, and cannot be inferred from any single external biomechanical descriptor, including foot-strike pattern.

These different levels of evidence should not be treated as equivalent: loading-rate and acceleration studies describe external or segmental impact-related indicators; modeling studies estimate structure-specific internal loading; and prospective studies or clinical trials reporting injury outcomes are needed to evaluate associations with, or effects on, running-related overuse injury occurrence [6, 7, 9, 11].

#### Theoretical Perspective: Impact as Adaptive Regulation

The preceding evidence indicates that impact-related measures should not be interpreted solely as indicators of mechanical harm. Nigg’s adaptive frameworks instead conceptualize running impact as one component of a dynamic control system. In their seminal work, Nigg and Wakeling [97] introduced the muscle-tuning paradigm, proposing that specific features of the impact signal—particularly its timing and frequency content—may contribute, through prior exposure and feedforward regulation, to anticipatory muscle activation that dampens soft-tissue vibrations and stabilizes limb motion before ground contact. This mechanism should not be interpreted as an immediate neuromuscular response triggered by the same impact event.

Extending this adaptive perspective, subsequent work advanced the concepts of a preferred movement path and a comfort filter [98], suggesting that runners self-select coordination patterns that regulate joint kinematics and muscle activity in response to the combined mechanical demands of ground-reaction forces, including impact-related loading. Within this framework, impact forces are interpreted in relation to neuromuscular control, perceived comfort, and energetic cost, which together contribute to the emergence of stable and repeatable movement solutions within individual constraints [6, 45, 46]. Variability in impact mechanics may therefore reflect functional adaptability within individual constraints rather than a pathological deviation from an idealized pattern.

Collectively, these perspectives position impact-related mechanical information as one component of anticipatory regulation and self-organization across repeated steps, rather than as a direct or isolated cause of injury.

#### Integrative Perspective

The heel-strike belief illustrates how visually persuasive yet reductionist interpretations of biomechanical data can evolve into dogma. Foot-strike patterns can represent different adaptive movement strategies, each redistributing mechanical stress differently along the kinetic chain. This interpretation is consistent with predictive simulations showing that the foot-strike pattern identified as “optimal” depends on the cost function being minimized [49]. Across 44 tested cost functions, rearfoot striking emerged most frequently and was optimal for several objectives related to whole-body energy expenditure and peak joint contact forces, whereas non-rearfoot patterns were favored for other objectives, including more evenly distributed muscle loading and lower average joint contact forces [49]. Thus, no single foot-strike pattern can be considered optimal independently of the functional objective being considered. Recognizing pronounced rearfoot striking as a potentially viable movement solution should not be interpreted as recommending that clinicians deliberately accentuate heel striking. Its clinical relevance remains dependent on the runner’s symptoms, tissue-specific loading requirements, functional response, and task demands.

This broader interaction is also illustrated by recent within-subject evidence showing that the biomechanical factors associated with changes in running economy differ among runners and footwear conditions [88]. Although this evidence is not specific to foot-strike modification, it supports the principle that the implications of a biomechanical characteristic emerge from interactions among the runner, the task, and the equipment rather than from a single technical variable [43, 44].

Rather than prescribing a universally correct technique, clinicians and coaches should emphasize gradual load adaptation and individualized feedback. Targeted foot-strike modification may nevertheless be appropriate when supported by a plausible tissue-specific rationale and by the runner’s symptoms or functional response. Its benefit should be evaluated through changes in symptoms, tolerance, and function and, when feasible, relevant tissue-specific loading outcomes, rather than inferred from external impact metrics alone [6, 45, 46, 99].

### Belief 2: The Universal Cadence Rule

#### Definition and Historical Context

The second enduring belief asserts that there exists an ideal cadence of 180 steps/min, universally associated with optimal efficiency and reduced injury risk. This notion originated from observations of competitive distance runners [22], in whom an average cadence near 180 steps/min was reported. Over time, this descriptive value became a practical reference point and was incorporated into some gait-retraining approaches [100]. The development of wearable devices capable of providing real-time cadence feedback has further facilitated the monitoring and use of such numerical targets during running [101, 102]. However, treating 180 steps/min as a broadly applicable ideal may insufficiently account for the substantial inter-individual and contextual variability that characterizes human running, including differences related to speed, morphology, footwear, and task demands.

#### Empirical Evidence: Cadence is Broadly Variable and Speed-Dependent

Extensive empirical evidence demonstrates that cadence varies widely among endurance runners, typically ranging from 150 to 210 steps/min [33, 34, 39, 103–105]. Laboratory studies consistently show a U-shaped relationship between cadence and metabolic cost, with each runner exhibiting a distinct preferred cadence that minimizes oxygen consumption [31, 106, 107]. Deviating substantially from this preferred cadence—either slower or faster—tends to increase energetic demand and perceived exertion.

**Fig. 1:**
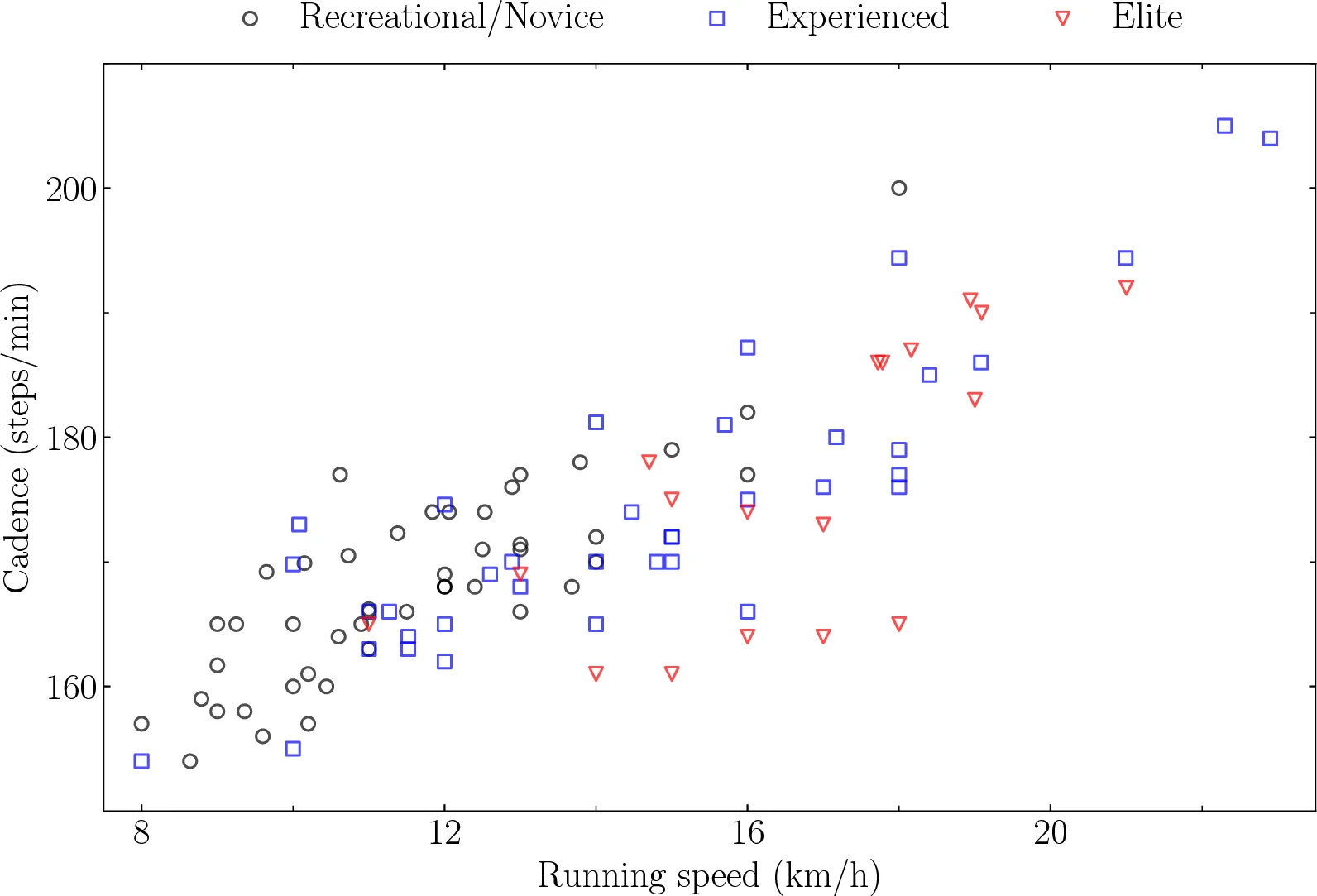
Cadence (steps/min) as a function of running speed (km/h) in recreational or novice (black circles), experienced (blue squares), and elite (red triangles) runners. The figure compiles group-level mean values reported in previously published studies [33, 34, 38–40, 51, 57, 105–108, 110–132] to provide a descriptive visualization of cadence across running speeds and runner populations. Each point represents a value extracted from an individual study or experimental condition. Running speed reflects the combination of cadence and step length; therefore, the figure is not intended to imply a linear or predictive relationship between cadence and running speed. No participant-level data were pooled

Cadence generally increases with running speed, as illustrated descriptively in Fig. 1. However, running speed results from the combination of cadence and step length, such that a given speed can be achieved through different combinations of these two variables. These combinations vary among runners [34], and the relative contributions of cadence and step length also change across the running-speed spectrum [108, 109]. At lower and moderate running speeds, increases in speed can be achieved predominantly through increases in step length, whereas at higher running speeds further increases depend increasingly on cadence [110]. Accordingly, the cadence–speed relationship should not be interpreted as universally linear, particularly across a broad range of running speeds. Figure 1 therefore compiles mean cadence values previously reported across multiple published studies involving recreational, experienced, and elite runners [33, 34, 38–40, 51, 57, 105–108, 110–132] to provide a descriptive visualization of cadence across running speeds and populations.

Cadence also varies with morphology, maturation, and environment. Longer legs or higher body mass have been associated with lower cadence in adult runners [34, 39], while adolescent runners at different stages of physical maturation exhibit differences in self-selected cadence even after accounting for running speed [41]. Pre-pubertal runners, for example, displayed higher cadences than mid- and post-pubertal runners, further illustrating that a single cadence target cannot be generalized across developmental stages. Footwear and surface properties also modify cadence dynamically [35–37, 40]. Barefoot running typically increases cadence by approximately 4–6% compared with shod running, driven by shorter strides, greater leg stiffness, and a more anterior foot-strike pattern [35, 36]. Minimalist footwear induces similar effects through heightened forefoot loading and reduced ground-contact times [37]. On compliant surfaces such as grass or sand, runners often increase cadence by 5–10% to maintain stability and effective force transmission despite lower surface stiffness [40]. Collectively, these findings confirm that cadence is not a fixed performance constant but a dynamic outcome of speed, individual characteristics, maturation, and environment.

#### Conceptual Clarification: The Limits of Prescriptive Cadence

The persistence of the 180 steps/min rule stems from its cognitive simplicity—it provides an easily quantifiable cue that appears grounded in biomechanics. However, this reductionism ignores the multifactorial nature of running economy, which depends on the interplay among stride length, limb stiffness, muscle–tendon elasticity, and neuromuscular coordination [31, 32, 133]. Cadence manipulations can acutely alter ground-reaction forces and joint kinetics, but such effects vary across individuals [23–26, 28]. A comprehensive meta-analysis confirmed that cadence adjustments can modify specific mechanical variables but found no consistent evidence linking these acute changes to improved performance or reduced overuse injury risk [2].

#### Theoretical Perspective: Cadence as a Constraint-Governed Outcome

Cadence should not be regarded as an independent driver of performance or injury risk but as a constraint-governed outcome of the broader locomotor system. Within the frameworks of the preferred movement path and comfort filter [45, 46, 98], runners tend to settle into cadence ranges that balance perceived effort, neuromuscular coordination, and task demands. Importantly, experimental work indicates that small, context-specific increases in cadence relative to an individual’s habitual pattern can reduce metabolic cost or redistribute tissue-level loading in some runners, consistent with the presence of a shallow optimum rather than a single fixed solution [32, 134]. From this perspective, modest cadence adjustments may be beneficial when applied judiciously, whereas externally imposed or uniform cadence prescriptions risk disrupting individual self-organization [31, 106, 107] and increasing energetic or mechanical costs.

Rather than seeking a universal ideal cadence, practitioners should recognize cadence as one adjustable manifestation of an integrated control strategy that evolves dynamically with speed, morphology, surface, and footwear. This systems-based perspective reframes cadence not as a target to be achieved, but as a product of adaptive coordination, shaped by the interaction of mechanical, sensory, and perceptual constraints.

#### Integrative Perspective

The endurance of the cadence rule reveals more about human cognition than biomechanics. By transforming a descriptive mean into a normative target, it offers the illusion of precision and control in a domain characterized by complexity and individual differences. This simplification persists because it satisfies scientific and coaching desires for simple, quantifiable guidance. Yet, as illustrated in Fig. 1, cadence generally increases with running speed and interacts with morphology and environment, leaving no empirical basis for a fixed optimal value. Treating cadence as a universal benchmark promotes conformity at the expense of functional adaptability.

Within an evidence-informed perspective, cadence should be viewed not as a fixed prescription but as a situated variable emerging from the interaction between runner, task, and environment [43, 44]. Adjustments to cadence can be valuable tools for managing specific mechanical or clinical issues, but optimization arises through individualized adaptation—not through numerical conformity. Recognizing cadence as a manifestation of self-organization under constraint allows practitioners to move beyond reductionist dogma toward a nuanced, context-sensitive understanding of running efficiency.

### Belief 3: The Cadence Prescription

#### Definition and Historical Context

The belief that increasing cadence—typically by 5–10% while maintaining running speed—reduces mechanical loading and injury risk underpins many gait retraining protocols [3, 135]. It originated from early laboratory studies showing that acutely increasing cadence at a fixed speed could reduce selected internal loading measures (e.g., patellofemoral joint force), external loading metrics (e.g., peak vertical force, braking impulse, loading rate), and muscle-activation patterns [23–26, 28, 136–138]. These short-term mechanical changes encouraged the adoption of cadence increases as generalized corrective strategies. These effects were readily observable, easy to cue, and appeared biomechanically intuitive, facilitating their translation into applied practice. However, subsequent syntheses integrating biomechanical and clinical perspectives have demonstrated that these effects are predominantly short-term, with limited evidence for sustained biomechanical adaptation or injury risk reduction [2, 4, 5]. This historical context motivates closer examination of the mechanisms underlying cadence-related loading changes.

#### Empirical Evidence: Contextualizing Cadence-Related Load Changes Through Stance–Flight Organization

Across studies, cadence manipulations can acutely alter internal and external loading, although the magnitude and direction of these responses vary between runners and may involve compensations elsewhere in the kinetic chain [24, 26, 28]. Importantly, cadence does not characterize how each stride is temporally distributed between stance and flight. Consequently, some mechanical responses observed during cadence manipulation may also reflect concurrent changes in stance–flight organization. DF provides a temporal descriptor through which this possibility can be examined. Under level, slow-to-submaximal running conditions, several studies have reported stronger associations between DF and selected vertical loading measures than between cadence and these same outcomes [57, 61–64, 139]. These findings do not establish DF as an alternative universal determinant or intervention target; rather, they indicate that stance–flight organization should be considered when interpreting mechanical responses attributed to cadence.

Much of the cadence-focused literature has not quantified DF, leaving uncertainty as to whether reported reductions in internal and vertical loading result from cadence itself, from concurrent changes in stance–flight organization, or from both [111, 138, 140–146]. Among studies that measured DF, increases in cadence were systematically accompanied by higher DFs [23, 25, 110, 114, 147, 148]. This co-variation suggests that some loading and muscle-activation changes associated with cadence manipulation may be partly mediated by concurrent changes in temporal organization. However, cadence and DF are not obligatorily coupled. Nijs, Roerdink and Beek [58] demonstrated that verbal instructions targeting stance or flight time can modify DF without clear concomitant changes in cadence during treadmill running at a given speed. Their subsequent field-based study showed that cadence and DF can also be manipulated during overground running, although changes in speed and the non-targeted temporal variable may occur when either variable is manipulated in isolation [59]. Evidence from our group further demonstrates that cadence can be manipulated across a functional range without systematic changes in DF [60]. Together, these findings indicate that cadence and DF are related but distinguishable temporal descriptors whose coupling depends on the task and mode of manipulation. DF should therefore be considered as a complementary descriptor when interpreting cadence-related mechanical responses, rather than as a predetermined target for modification.

#### Conceptual Clarification: External Versus Internal Load, and Redistribution

The relevance of stance–flight organization becomes particularly important when moving from external mechanical measures to internal tissue loading. Running-related overuse injuries may develop when cumulative internal tissue loading exceeds the tissue’s current capacity to tolerate, recover from, and adapt to repeated loading [149, 150]. Despite this, external kinetic measures such as peak vertical ground reaction force and vertical average loading rate are often used as surrogates for internal stress, even though their associations with muscle, tendon, and joint loading are inconsistent and generally weak [7, 8, 11, 141, 150, 151].

Within this context, DF can help characterize how force is distributed over stance relative to the complete stride. Experimental and modeling studies indicate that manipulation or variation of DF can influence both external and internal loading [63, 64]. Recent experimental work independently manipulating DF and cadence showed that increasing DF reduced peak joint reaction forces and extensor moments across the ankle, knee, and hip, with the exception of hip flexor moments, which increased [63]. Cadence produced weaker and more variable effects on internal loading [63]. Higher DF has also been associated with reduced peak joint reaction forces, muscle–tendon loading, and internal bone and joint stresses across multiple lower-limb structures [63, 64]. These findings provide mechanistic evidence that temporal stance–flight organization contributes to musculoskeletal load distribution under the level, submaximal running conditions studied. Consistent with these findings, the aforementioned high-mileage runner displayed a higher DF than matched controls (41% vs. 34%), together with lower external loading-related measures [83]. Although observational, this case further illustrates the potential contribution of stance–flight organization to external loading.

Importantly, these results should not be interpreted as evidence that increasing DF is generally desirable. The observed effects are structure-specific and are derived predominantly from acute biomechanical and modeling studies. Clinical evidence does not support a uniformly beneficial direction of stance-time or DF modification. During rehabilitation for exercise-related lower leg pain, improved patient-reported outcomes have been associated with reduced DF [152]. In a separate case–control study, runners with exercise-related lower leg pain displayed longer absolute contact times than healthy runners after adjustment for running pace [153]. However, DF was not reported in that study, and step rate and flight ratio did not differ between groups. Conversely, shorter stance time has been identified as a potential risk factor for bone stress injury in collegiate runners [154]. Contact time and DF are related but non-equivalent variables [129], and these observational or multicomponent rehabilitation findings do not establish an independent causal effect. Collectively, they reinforce that temporal modifications redistribute loading rather than uniformly reducing it, and that their clinical relevance depends on the tissue, condition, runner, and task.

#### Theoretical Perspective: Spatiotemporal Control and Constraint-Driven Adaptation

Running coordination arises from the interaction of mechanical, neuromuscular, and environmental constraints. Within this system, cadence and the temporal distribution of stance and flight emerge together as features of the runner’s coordination strategy [45, 47]. Cadence and DF can therefore provide complementary information about running organization without implying that either constitutes an isolated mechanical control lever [48].

Within the level, slow-to-submaximal running conditions studied, increases in relative stance duration and/or reductions in flight time can reduce selected vertical and modeled internal loading measures by distributing the ground-reaction impulse differently over time [57, 61–64]. DF provides a convenient means of describing these changes. Whether these relationships extend to steep uphill or downhill running, high-velocity elite training, or sprinting remains uncertain.

These adaptations reflect the capacity of the locomotor system to reorganize coordination in response to energetic, mechanical, sensory, and perceptual constraints [30, 32]. This interpretation aligns with the preferred movement path and comfort filter paradigms [45, 46, 98], which propose that runners tend to select patterns that minimize effort within their individual constraints. Accordingly, gait retraining should not assume that either cadence or DF needs to move in a predetermined direction. Instead, manipulation of task constraints may be used to explore alternative coordination solutions when a specific mechanical, symptomatic, functional, or performance objective justifies doing so.

#### Integrative Perspective

The cadence prescription belief illustrates how an acute biomechanical response can be translated into a generalized intervention despite uncertainty regarding the mechanism responsible for that response and its longer-term clinical relevance. Increasing cadence can modify selected mechanical variables, but cadence changes may occur alongside changes in stance–flight organization, making it difficult to attribute all observed effects to cadence alone when temporal variables are not concurrently quantified.

DF is useful in this context because it provides complementary information about the temporal organization of the stride. It should therefore be viewed primarily as an interpretive descriptor that can help clarify cadence-related mechanical responses. In selected circumstances, DF may also be explored as an intervention variable when altering stance–flight organization has a plausible tissue-specific or functional rationale. However, current evidence does not yet support DF as an established stand-alone gait-retraining strategy or a universally preferable target [5].

Accordingly, the evidence reviewed here does not support replacing a universal cadence prescription with a universal DF prescription. Instead, cadence manipulation should be considered according to the runner’s clinical or performance objective, while concurrent changes in stance–flight organization can be monitored when they help interpret the mechanical response. The success of any intervention should ultimately be evaluated through symptoms, tolerance, function, performance, and, where appropriate, tissue-specific loading rather than through achievement of a predetermined cadence or DF.

## Conclusions—Practical Implications and Future Directions

### From Prescriptions to Principles

Across the three beliefs examined, the available evidence does not support a universal foot-strike pattern, a universal cadence value, or a universally beneficial direction of cadence modification.

Foot-strike pattern and cadence represent context-dependent features of running coordination, while DF can provide complementary information about the temporal distribution of stance and flight, particularly when interpreting mechanical responses to cadence modification. None of these measures should be interpreted in isolation from individual structure, motor control, task demands, symptoms, and training background. Their interpretation should therefore remain specific to the runner and context rather than be generalized to all dimensions of running technique [31, 46–48, 155, 156].

### Individualized Assessment and Decision-Making

Before modifying running mechanics, practitioners must determine whether change is warranted [157]. Effective assessment distinguishes natural variability from potentially maladaptive loading using three diagnostic filters:

- **Symptom relevance**—is there a plausible mechanical contribution to pain or dysfunction?
- **Load tolerance**—are tissues operating near or beyond their adaptive capacity?
- **Adaptation potential**—does the runner possess the physical and perceptual capacity required for motor change?

Assessment should integrate quantitative measures relevant to the clinical or performance question with qualitative observations such as comfort, effort, and movement fluency. Cadence, force profiles, loading rate, and, when relevant, DF can provide complementary information about running organization, but none should be interpreted independently as an indicator of technique quality. Mapping runners along the cadence–DF spectrum [48] or the terrestrial–aerial continuum [122, 157] can provide a broader functional representation of the running pattern without imposing normative targets.

### Evidence-Informed Principles for Technique Modification

The recommendations below are supported by different levels of evidence. Direct clinical evidence is available for selected gait-retraining interventions, including cadence-based approaches in runners with patellofemoral pain [15, 125, 158], as well as for individualized wearable-based gait modification [159]. Evidence concerning foot-strike modification and DF is predominantly mechanistic, observational, or modeling-based and therefore supports tissue-specific hypotheses rather than broadly established clinical prescriptions [1, 30, 57, 61–64, 77, 93]. Condition-specific clinical and observational findings remain limited and do not support a universally beneficial direction of stance-time or DF modification [152–154]. Principles concerning constraint manipulation, comfort, and feedback withdrawal are additionally informed by motor-learning and theoretical frameworks and require further direct clinical evaluation [43–47, 98, 160, 161].

- **Determine whether modification is warranted.** Preserve habitual running mechanics when no symptom-related, functional, or performance-related rationale for change is present.
- **Define the tissue-specific or functional objective.** Identify the symptomatic structure, task limitation, and intended load redistribution before selecting a biomechanical variable.
- **Select the variable according to the available evidence.** Cadence may be tested when supported by condition-specific clinical evidence, whereas foot-strike modification should be considered only when a plausible tissue-specific rationale is present. When cadence or other spatiotemporal variables are modified, DF may additionally be monitored to determine whether changes in stance–flight organization accompany the intervention. In selected cases, DF itself may be explored when modifying stance–flight organization has a specific mechanistic rationale, although evidence supporting this application remains predominantly biomechanical. The direction and magnitude of any modification should be individualized. Current evidence does not support prescribing universal targets or giving cadence, foot-strike pattern, or DF automatic precedence.
- **Respect progressive adaptation.** Structural and neuromuscular remodeling require time; abrupt changes may merely redistribute stress [84, 86].
- **Use feedback strategically.** Targeted feedback can facilitate short-term modification of cadence or other spatiotemporal characteristics [60, 161], but should subsequently be reduced when the aim is to establish intrinsic control of the adapted movement solution.
- **Integrate comfort and perception.** Perceived comfort may help assess whether a modification is compatible with the runner’s preferred movement path and current tolerance [46, 98]. Persistent discomfort may indicate that the intervention is poorly tolerated or produces an unfavorable reorganization.
- **Embed technique within holistic load management.** Mechanical change interacts with training volume, footwear, surface, and recovery. Coordinated load management remains the primary safeguard against injury [4].

### Translational Guidance for Clinicians, Coaches, Researchers, and Industry and Research and Development

#### Clinicians

This paradigm reframes gait retraining from a corrective to an adaptive process [157]. The aim is sustainable, tissue-appropriate load distribution and motor adaptability, supported by environmental or task constraints rather than rigid form rules. Success should be judged by symptoms, tolerance, function, and task-specific performance rather than by the achievement of a predetermined value [162].

A practical decision pathway begins by determining whether modification is required. In an asymptomatic runner without a relevant functional or performance limitation, habitual running mechanics should generally be preserved. When modification is indicated, the symptomatic tissue and intended mechanical or functional objective should first be identified. Cadence may be tested when supported by condition-specific clinical evidence, particularly in runners with patellofemoral pain [15, 125, 158]. Foot-strike pattern may be explored when the habitual pattern plausibly contributes to loading of the symptomatic structure and when a gradual trial produces a favorable symptomatic and functional response [1, 30, 77, 84, 87, 88, 93]. When these or other spatiotemporal modifications are implemented, DF can additionally be monitored to characterize accompanying changes in stance–flight organization. Direct manipulation of DF may be considered in selected situations where such temporal reorganization has a plausible tissue-specific rationale, but current clinical evidence is insufficient to recommend a predetermined direction of change [57, 61–64, 152–154]. In all cases, the intervention should be retained only if it improves symptoms, tolerance, function, or task-specific performance without producing adverse effects elsewhere.

#### Coaches

Coaching practice should emphasize flexibility and experiential learning. Technique should evolve through iterative exploration, using individualized cues and feedback grounded in perceptual experience [160]. Training diversity—varying surfaces, footwear, and speeds—broadens the runner’s movement repertoire and supports robust, adaptable coordination.

#### Researchers

Research should first determine whether gait-retraining interventions targeting foot-strike pattern or cadence produce sustained improvements in symptoms, function, overuse injury occurrence, or performance beyond usual care and training-load management. Comparative longitudinal studies should identify which runners respond to each intervention and whether the appropriate variable, direction, and magnitude of modification depend on the symptomatic tissue, baseline coordination, running speed, gradient, footwear, fatigue, or performance level. Studies examining cadence-based interventions should additionally quantify changes in stance–flight organization, including DF where appropriate, to determine whether these changes contribute to the observed mechanical or clinical response. Direct manipulation of DF should also be investigated experimentally to establish whether it provides clinically meaningful benefits in specific populations or conditions. Individualized or constraint-based interventions should also be compared with fixed prescriptions and usual-care approaches.

To clarify the translation from biomechanical modification to clinical benefit, studies should measure both the prescribed and achieved changes in running mechanics, alongside external loading, estimated or directly assessed internal tissue loading, symptoms, function, training exposure, adherence, adverse effects, and retention after feedback withdrawal. When cadence is manipulated, studies should report concurrent changes in contact time, flight time, and DF, allowing cadence-specific effects to be distinguished from broader changes in temporal organization. Both group-level effects and individual response patterns should be reported. Population estimates can identify average tendencies, whereas repeated-measures and n-of-1 approaches can determine whether a response is consistent and clinically meaningful within a given runner. Prospective, individualized designs are therefore needed to identify who benefits from a given intervention and under which conditions [163]. Studies specifically examining stride-to-stride variability should additionally consider its temporal structure rather than variability magnitude alone [53].

Research should also determine whether weighted combinations of spatiotemporal and kinematic variables provide reliable and interpretable representations of running technique beyond isolated parameters [164, 165]. Such composite measures should be validated across speeds, gradients, surfaces, footwear conditions, runner populations, and clinical or performance outcomes, and compared with broader coordination representations such as the cadence–DF spectrum and terrestrial–aerial continuum [48, 122, 157].

#### Industry and Research and Development

Technology development should move beyond providing isolated biomechanical metrics toward systems that support context-specific interpretation and individualized decision-making. Current wearable devices can estimate spatiotemporal variables and selected external or internal loading outcomes outside the laboratory, particularly when sensor data are combined with machine-learning or biomechanical modeling approaches [11, 166–168]. However, improving measurement accuracy alone may be insufficient if the resulting variables are interpreted as universal indicators of running quality or injury risk.

Future systems may benefit from multimodal sensing that combines temporal, kinetic, kinematic, physiological, and contextual information to better characterize the interaction between the runner, task, and environment. For example, simultaneously monitoring cadence, contact and flight characteristics, running speed, gradient, and relevant loading estimates could help distinguish whether an observed mechanical response arises from the targeted variable or from broader changes in running organization. Modeling and data-driven approaches may further assist in identifying multidimensional running patterns that cannot be captured by isolated variables [169, 170].

Technology may also support hybrid models in which automated real-time feedback is combined with periodic interpretation by clinicians or coaches. Wearable-based individualized feedback has already shown potential to modify running behavior and reduce injury rate and severity without compromising performance [159], but future platforms should prioritize interpretable feedback, progressive withdrawal of external guidance, and integration with symptoms, training exposure, and functional outcomes rather than continuously enforcing predetermined biomechanical targets. Industry and R&D efforts should therefore focus not only on sensing accuracy, but also on validating which combinations of variables meaningfully inform clinical and performance decisions across different runners and running conditions.

### Toward a Pluralistic Paradigm

The evidence reviewed here supports interpreting foot-strike pattern and cadence as context-dependent features of running coordination rather than as universally correct or incorrect techniques. Temporal descriptors such as DF can provide additional information about how these movement solutions are organized and may help explain some of their mechanical consequences.

A further consideration is the aesthetic perception of running form. Movement patterns that depart markedly from conventional or visually preferred running styles may be perceived as inefficient or “poor” technique even when they represent viable solutions for a given runner and task. For example, a pronounced increase in DF may reduce or eliminate flight time and, at sufficiently high values, result in grounded running characterized by alternating single and double support without an aerial phase [171]. Such a pattern may reduce selected external and internal loading measures [172] while appearing less conventionally “athletic” or aesthetically desirable. Importantly, the absence of a flight phase does not necessarily imply a transition to walking, as grounded running retains mechanical characteristics of running despite continuous ground contact [171]. Aesthetic judgments should therefore be distinguished from evidence of dysfunction, excessive tissue loading, impaired performance, or adverse clinical outcomes. Within a pluralistic framework, the visual appearance of a running pattern may be relevant to the runner’s preferences or goals, but should not in itself determine whether modification is warranted.

Early insights noted that running form emerges from interactions among foot placement, arm swing, posture, and stride dynamics [173]. Contemporary evidence shows that runners self-optimize within their constraints, achieving similar energetic efficiency through diverse styles [31, 45, 50–52, 156, 174]. In this context, functional adaptability reflects the system’s capacity to redistribute mechanical load, regulate sensory input, and adjust to changing task demands without overloading specific tissues. Rather than representing technical error, this adaptability may enhance robustness by allowing continuous adjustment within biological tolerance limits [30, 46]. The future of gait retraining therefore lies in cultivating adaptive coordination through targeted constraints, progressive feedback, and contextual practice.

For the beliefs examined here, progress requires replacing universal prescriptions with context-specific assessment. Clinicians should determine whether technique modification is warranted rather than assume that change is necessary, coaches should guide exploration rather than enforce fixed values, researchers should evaluate clinical and performance outcomes rather than infer benefit from acute biomechanical changes alone, and industry and R&D should develop technologies that support individualized interpretation rather than reinforce universal biomechanical targets. Cadence, foot-strike pattern, and complementary descriptors of temporal organization such as DF can then be interpreted according to the individual runner, task, and intended outcome.

## Data Availability

Not applicable.

## Abbreviation

DF: Duty factor
